# Dolutegravir Associated glycaemia Among Persons with HIV on Treatment at a Kenyan Referral Hospital

**DOI:** 10.24248/eahrj.v8i3.808

**Published:** 2025-01-30

**Authors:** Judith A Odenyo, George A Mugendi, David G Nyamu, Andew A Okiko

**Affiliations:** a Department of Pharmacology, Clinical Pharmacy and Pharmacy Practice, Faculty of Health Sciences, University of Nairobi, Nairobi; b Kenyatta, National Hospital, Nairobi, Kenya.

## Abstract

**Background:**

Dolutegravir-based antiretroviral therapy is a clinically proven treatment option for persons living with the human immunodeficiency virus. However, there is emerging clinical evidence that its use may result in hyperglycaemia, but there is limited data in Africa.

**Objectives:**

To determine the prevalence of dolutegravir-associated hyperglycaemia and its covariates among Persons Living with HIV on treatment in a tertiary teaching and referral hospital in Kenya

**Methods:**

A cross-sectional study was undertaken among adult patients who had been using dolutegravir-based regimens for at least 3 months at the comprehensive care centre in Kenyatta National Hospital. An interviewer-administered questionnaire was used to collect sociodemographic characteristics while clinical data (viral load, CD4 count) were abstracted from patients’ charts. Blood samples were taken to determine random blood sugar and glycated haemoglobin levels. Data were analysed using STATA^®^ statistical software. Associations between hyperglycaemia and patients’ sociodemographic and/or clinical data were determined as appropriate whereas the independent correlates of hyperglycaemia were determined through multivariable logistic regression at *P*≤.05.

**Results:**

We recruited 358 participants all on dolutegravir based therapy and predominantly female (62.0%). Median age was 444 (IQR 38 to 50) years. Prevalence of hyperglycaemia was 55.9%. Age above 40 years (aOR1.7; 95% CI, 1.1 to 2.7; *P*=.026), being overweight (aOR 1.7; 95% CI, 1.1 to 2.8; *P*=.026) and obesity (aOR 3.1; 95% CI, 1.7 to 5.5; *P*<.001) were associated with experiencing hyperglycaemia.

**Conclusion:**

Dolutegravir-based regimens are associated with hyperglycaemia among patients with advanced age and elevated body mass indices.

## BACKGROUND

The World Health Organization (WHO) recommends the adoption of antiretroviral regimens with high potency, lower incidences of adverse events, high genetic barrier to resistance, and improved efficacy across different populations. Consequently, dolutegravir (DTG) based highly active antiretroviral therapy (HAART) regimens were recommended as the preferred first and second-line treatment for all populations.^1^ Dolutegravir (DTG) is an antiretroviral (ARV) agent for the management of HIV-1 infection, in combination with other ARVs.

Dolutegravir (DTG), an integrase strand transfer inhibitor (INSTI), possesses unique properties such as un-boosted daily dosing, a higher genetic barrier to resistance and minimal cross-resistance with the first generation INSTIs.^2^ The WHO supported the transition to DTG-based regimens, to optimize ART, especially in regions with suspected pre-treatment failure of more than 10% to either efavirenz or nevirapine, as commonly seen in Southern and Eastern Africa.^3^ Prolonged use of ART is associated with the risk of developing metabolic syndromes that can manifest with hyperglycaemia, depending on the ART regimen used. Reports associating the use of DTG with the development of hyperglycaemia are available.^4,5^ However, there is a dearth of information on the prevalence of hyperglycaemia associated with DTG use in Kenya among Persons Living with HIV (PLHIV). Regionally, reports of the association between DTG and hyperglycaemia have been made, ranging from case series in Ethiopia, a case-control study in Uganda to synthesis of pharmacovigilance reports in Eswatini.^6–9^

Based on the gaps in the patient care process, we designed and implemented an exploratory hypothesis generating cross-sectional study to determine the prevalence of and correlates of hyperglycaemia among patients on DTG-based regimens in a resource-limited setting.

## METHODOLOGY

### Study Design and Site

An analytical cross-sectional study was undertaken among adult patients undergoing long-term antiretroviral therapy (≥ 3 months) at the Comprehensive Care Centre (CCC) of Kenyatta National Hospital (KNH) between the months of July and September 2020.

### Study Population

The study population comprised all HIV patients enrolled and on follow-up at KNH CCC, on DTG-based therapy, for at least 3 months.

### Inclusion and Exclusion Criteria

The patients were either treatment naïve or switched from a previous regimen to a DTG-based regimen, and had come to the clinic for review or medication re-fill. In addition, each one were to have willingly agreed, through written consent, to participate in the study and proceeded to the clinic laboratory for sample collection for both random blood sugar (RBS) and glycosylated hemoglobin (HbA1c) measurements. All patients who had documented or reported diagnosis of diabetes, those on chronic use of corticosteroids or pregnant during the study were excluded from the study.

## CASE DEFINITIONS

Cases were defined as laboratory reports of hyperglycaemia, RBS and HbA1c measurements, based on KNH reference ranges. Hyperglycaemia diagnosis was made by HbA1c measurement ≥ 6.1% and/or a random blood glucose ≥7.2 mmol/L.

### Sample Size Estimation and Sampling Method

The main outcome variable was the diagnosis of hyperglycaemia. Due to a lack of published data on the exact prevalence of DTG-associated hyperglycaemia in our region at the time, the prevalence of protease inhibitors associated with hyperglycaemia,^10^ was used as a proxy to estimate the sample size of 358 participants using the Cochran formula.^11^ A consecutive sampling technique was employed to reach the calculated sample size. Every participant who met the inclusion criteria and was willing to participate was enrolled in the study.

### Ethical Considerations

Ethical approval was sought from the Kenyatta National Hospital/University of Nairobi-Ethics and Research Committee (KNH/UoN-ERC) reference KNH-ERC/A/212. Eligible participants were taken through the consenting process and those who voluntarily accepted to participate in the study were requested to sign the informed consent document.

### Statistical Analysis

Data from the study questionnaires were entered into a password-protected database. Data analysis was done using Stata version 13 software (Stata Corp, USA). The Shapiro Wilk test was performed on all continuous variables to check for normality of data distribution. The association between predictor variables (age, sex, and Body Mass Index (BMI)) and hyperglycaemia was tested using chi-square or fisher’s tests for normally distributed variables. Inferential statistics were performed to compare the distribution of variables across participants with hyperglycaemia versus those without hyperglycaemia. Bivariate and multivariable logistic regression analyses were used to determine the independent covariates of hyperglycaemia development in the study population, and also to adjust for potential confounding factors. Age, sex, BMI, level of physical activity, presence of comorbidities, duration of DTG therapy and type of DTG regimen were some of the patient variables included in the regression model.

## RESULTS

A total of 358 participants were enrolled. Most of the participants were females (n=222, 62.0%) and the median age was 44 (IQR, 38 to 50) years with a range of 18 to 72 years. The median BMI was 25.8 (IQR, 22.6 to 29.6) kg/m^2^. More than 50% of the participants were either overweight (n=127, 35.5%) or obese (n=81, 22.6%) (Table 1).

**Table 1.** Baseline Characteristics of the Study Population

Variable	Frequency (n)	Percentage (%)
Age (years)		
18–40	116	32.4
41–60	226	63.1
>60	16	4.5
Sex		
Male	136	38.0
Female	222	62.0
Marital status		
Single	138	38.5
Married	220	61.5
Education		
Informal	8	2.2
Primary	69	19.3
Secondary	143	39.9
Tertiary	138	38.5
Employment status		
Unemployed	73	20.4
Self-employed	122	34.1
Formal	120	33.5
Informal	43	12.0
BMI (Kg/m^2^)		
<18.5	10	2.8
18.5–24.9	140	39.1
25.0–29.9	127	35.5
>=30.081	22.6	
Level of physical activity		
None	9	2.5
Minimal	72	20.1
Moderate	140	39.1
Active	137	38.3
Smoking status		
No	352	98.3
Alcohol use		
No	331	92.5
Viral load		
Low VL (<1000 copies/ml)	317	88.5
High VL (>1000 copies/ml)	15	4.2
No VL done	26	7.3
CD4+ count (cells/mm^3^)		
Low CD4+ (<200 cells/mm^3^)	38	10.6
High CD4+ (>200 cells/mm^3^)	176	49.2
No CD4+ count done	144	40.2
Comorbidity		
None	348	97.2
Arthritis	1	0.3
Hypertension	5	1.4
Tuberculosis	2	0.6
Hepatitis	1	0.3
Asthma	1	0.3

Most participants (n=317, 88.5%) were virally suppressed (HIV-1 RNA<1000 copies/ml) prior to being switched to DTG, with only 4.2% of the study population having viral loads (VL)> 1000 copies/ml but almost half had CD4 counts greater than 200 cells/mm^3^ (n=176, 49.2%).

**Figure 1.**
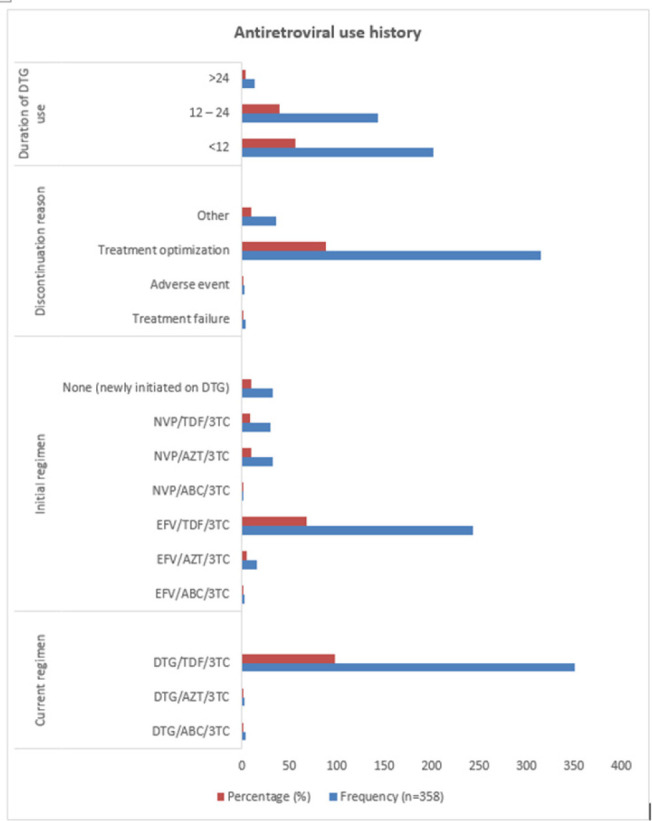
History of Antiretroviral Therapy Among the Participants

Most patients (n=351, 98%) had had their regimens modified to include DTG, with only 33 (9.2%) being directly initiated on the drug after testing. The main reason for treatment modification was the optimization of therapy (n=315, 88.0%) (Figure 1).

Patients were assessed and their glycaemic status evaluated via both RBS and HbA_1_c measurements. The cut-off points for hyperglycaemia were RBS≥7 mmol/L and HbA1c≥6.1%. Eight (2.2%) and 199 (55.6%) participants were found to be hyperglycaemic via RBS and HbA_1_c, respectively (Table 2).

**Table 2.** Tests for Hyperglycemia for the Study Participants

Hyperglycaemia test	Frequency (n=358)	Percentage (%)
RBS (mMol/L)		
Normoglycemia (3.6–7.1)	350	97.8
Hyperglycemia (≥7.2)	8	2.2
HbA1c (%)		
Normoglycemia (3.5–6.0)	159	44.4
Hyperglycemia (≥6.1)	199	55.6

Key: - RBS (Random Blood Sugar); HbA1c (Glycosylated hemoglobin A)

Hyperglycemia was found to be significantly associated with age, sex and a BMI>25 at bivariable logistic regression analysis (Table 3). The odds of developing hyperglycaemia for those above 40 years was 1.7 (95% CI, 1.1 to 2.7, *P*=.014) times higher compared to individuals under 40. Females had 1.6 (95% CI, 1.1 to 2.5, *P*=.029) times higher odds of developing hyperglycaemia compared to males. Individuals with BMI>25 had 1.8 (95% CI, 1.1 to 2.9, *P*=.017) times the odds of developing hyperglycaemia compared to persons with a normal BMI. Obesity raised the odds of having hyperglycaemia to 3.1 (95% CI, 1.7 to 5.6, *P*<.001) times that of persons with a normal BMI. At multivariable logistic regression analysis, age (aOR 1.7, 95% CI, 1.1 to 2.7, *P*=.026), being overweight (aOR 1.7, 95% CI, 1.1 to 2.8, *P*=.026) and obesity (aOR 3.1, 95% CI, 1.7 to 5.5, *P*=<.001) were independently associated with the occurrence of hyperglycaemia.

**Table 3.** Univariate Analysis of the Sociodemographic Characteristics and Glycemic Status of the Participants

Variable	Hyperglycemic (n, %)	Normoglycemic (n, %)	*P Value*
Age			
18–40	54 (15.1)	62 (17.3)	.023
>40	145 (40.5)	97 (27.1)	
Sex			
Male	65 (18.2)	71 (19.8)	.027
Female	134 (37.4)	88 (24.6)	
BMI			
<18.5	3 (0.8)	7 (1.9)	<.001
18.5–24.9	64 (17.9)	77 (21.5)	
25.0–29.9	75 (20.9)	51 (14.2)	
>=30.0	57 (15.9)	24 (6.7)	
Physical activity			
None	7 (1.9)	2 (0.6)	.703
Minimal	40 (11.1)	32 (8.9)	
Moderate	83 (23.2)	57 (15.9)	
Active	69 (19.3)	68 (19.0)	
Smoking			
No	194 (54.2)	158 (44.1)	.232
Yes	5 (1.4)	1 (0.3)	
Alcohol			
No	186 (52.0)	145 (40.5)	.543
Yes	13 (3.6)	14 (3.9)	
Current regimen			
DTG/ABC/3TC	3 (0.8)	1 (0.3)	.292
DTG/AZT/3TC	3 (0.8)	0	
DTG/TDF/3TC	193 (53.9)	158 (44.1)	
Duration of DTG therapy (months)			
< 12	103 (28.8)	99 (27.7)	.126
12–24	87 (24.3)	56 (15.6)	
>24	9 (2.5)	4 (1.1)	
Comorbidity			
No	166 (43.4)	142 (39.7)	.149
Yes	33 (9.2)	17 (4.7)	

**Table 4.** Bivariable and Multivariable Logistic Regression Analysis of the Association Between the Presence of Hyperglycemia and Patient Related Factors

Variable	Bivariable analysis		Multivariable analysis	
	cOR (95% CI)	*P Value*	aOR (95% CI)	*P Value*
Age				
18–40	Reference			
>40	1.7 (1.1–2.7)	.014	1.7 (1.1–2.7)	.026
Sex				
Male	Reference			
Female	1.6 (1.1–2.5)	.029		
BMI				
≤24.9	Reference			
25.0–29.0	1.8 (1.1–2.9)	.017	1.7 (1.1–2.8)	.026
≥30.0	3.1 (1.7–5.6)	<.001	3.1 (1.7 – 5.5)	<.001
Level of physical activity				
None/Minimal	1.1 (0.7–1.8)	.657		
Moderate/Active	Reference			
Smoking status				
Yes	4.0 (0.5–34.8)	.206		
No	Reference			
Alcohol use status				
Yes	0.7 (0.3–1.6)	.403		
No	Reference			
Comorbidity				
Yes	1.6 (0.9–3.1)	.122		
No	Reference			
DTG-based ART duration of use (months)				
<12	Reference			
12–24	1.5 (1.0–2.4)	.053		
>24	2.2 (0.6–7.3)	.211		
Current regimen				
DTG/ABC/3TC	Reference			
DTG/AZT/3TC	–			
DTG/TDF/3TC	0.4 (0.04–4.0)	.444		

## DISCUSSION

In this cross-sectional study, we enrolled 358 patients who had been on DTG-based regimens for at least 3 months to determine the prevalence of hyperglycaemia and its associated factors. Two hundred patients (55.9%) were found to be hyperglycaemic. Age above 40 years and BMI above 25kg/m^2^ were found to be independently associated with the presence of hyperglycaemia.

Data on the prevalence of DTG-induced or associated hyperglycaemia is rather sparse. Regionally, prevalence data ranging from as low as 0.47% to as high as 73.5% have been reported in Uganda.^6,9,12^ The differences in the burden of hyperglycaemia could be attributed to methodology; sample size, duration of follow up and study design. The prevalence of DTG-induced hyperglycaemia reported from pharmacovigilance data collected over a 12-year period in the US is 0.09%, further displaying the heterogeneity of findings.^13^ Of interest though, is a finding from a cohort study in Uganda where the authors report that there is no worsening of glucose metabolism throughout follow-up.^14^ The cohort was significantly younger with a median age of 31 years, and it was predominantly females. Approximately two-thirds had a normal BMI, while other factors are less associated with a risk of incident hyperglycaemia on DTG exposure.

Patients older than 40 years were more likely to have hyperglycaemia compared to those under 40 years. This finding is corroborated by reports indicating that older age is not only associated with adverse drug events from antiretroviral therapy, but that DTG use among this population increases the likelihood of having hyperglycaemia after some period of use.^9,15^ However, a study among Ethiopian immigrants in Israel concluded that the prevalence of hyperglycaemia and its complications in patients on HAART was age-independent, but detectable in patients <42 years.^16^ Our postulation regarding this finding is that older patients, above the age of 40 years are at risk of type 2 diabetes, coupled with the mechanism of action of DTG that tends to result in a possibility of insulin resistance multiplies the chance of incident hyperglycaemia.

Of the patients identified as experiencing hyperglycaemia in our study, slightly more than a third were either overweight or obese. Similar findings were reported in the randomized controlled trials in South Africa (ADVANCE study) and Cameroon (NAMSAL study) where patients on DTG-based regimens were either clinically obese or clinically overweight.^17,18^ A contrary finding in a case-control study in Uganda reported weight loss among the majority of patients, although their definition of cases was restricted mainly to symptomatic patients and/or those on antidiabetics, which could be partly responsible for the observed weight loss.^6^ The mechanism of DTG-induced increase in weight is unclear, but a report of a study suggests that exposure to the drug is associated with increased adipocyte differentiation, with enhanced expression of markers associated with lipid storage.^19^

In the present study, overweight and obese patients were more likely to have hyperglycaemia when compared with patients with normal BMI. Namara *et al*. reported that having a normal BMI was protective against developing DTG induced hyperglycaemia.^9^ Being overweight or obese is an established risk factor for developing type 2 diabetes mellitus, primarily as a result of insulin resistance due to the exorbitant expansion of white adipose tissue that impairs glucose transport resulting in insulin resistance.^20^

Coupled with the mechanism of action of DTG that also ultimately leads to insulin resistance, overweight and obese patients’ risk of developing hyperglycaemia is multiplied.

### Study Limitations and Strength

Our study was not without limitations. The glycaemic status of more than 90% of the studied population was not documented at the initiation of the DTG regimen. This, in addition to the cross-sectional design of the study, limited our ability to determine causality. However, we had a large sample size which meant our study was adequately powered to detect small differences within the study population.

## CONCLUSIONs AND RECOMMENDATIONS

The prevalence of hyperglycaemia in individuals on DTG-based regimens is high, especially among those above 40 years who are likely to have exceeded the normal BMI. Regular glucose monitoring for patients on DTG especially those with the identified risk factors should be considered. Large prospective studies are needed to ascertain causality of hyperglycaemia and DTG-based regimens.
